# Identification of Microorganisms Dwelling on the 19th Century Lanna Mural Paintings from Northern Thailand Using Culture-Dependent and -Independent Approaches

**DOI:** 10.3390/biology11020228

**Published:** 2022-01-31

**Authors:** Nattaphon Suphaphimol, Nakarin Suwannarach, Witoon Purahong, Churdsak Jaikang, Kamonpan Pengpat, Natthawat Semakul, Saranphong Yimklan, Surachai Jongjitngam, Saiklang Jindasu, Sathaporn Thiangtham, Panuwan Chantawannakul, Terd Disayathanoowat

**Affiliations:** 1 Department of Biology, Faculty of Science, Chiang Mai University, Chiang Mai 50200, Thailand; nattaphon.supp@gmail.com (N.S.); panuwan.c@cmu.ac.th (P.C.); 2 Research Center of Microbial Diversity and Sustainable Utilization, Chiang Mai University, Chiang Mai 50200, Thailand; suwan.462@gmail.com; 3 Department of Soil Ecology, UFZ-Helmholtz Centre for Environmental Research, 06120 Halle (Saale), Germany; 4 Toxicology Section, Department of Forensic Medicine, Faculty of Medicine, Chiang Mai University, Chiang Mai 50200, Thailand; churdsak.j@cmu.ac.th; 5 Department of Physics and Materials Science, Faculty of Science, Chiang Mai University, Chiang Mai 50200, Thailand; kamonpan.p@cmu.ac.th; 6 Department of Chemistry, Faculty of Science, Chiang Mai University, Chiang Mai 50200, Thailand; natthawat.semakul@cmu.ac.th (N.S.); Saranphong.Yimklan@cmu.ac.th (S.Y.); 7 Department of Thai art, Faculty of Fine arts, Chiang Mai university, Chiang Mai 50200, Thailand; surachai.j@cmu.ac.th; 8 The 7th Regional Office of Fine arts, Department of Fine Art, Ministry of Culture, Chiang Mai 50300, Thailand; klangjindasu@gmail.com (S.J.); thanw3@yahoo.com (S.T.); 9 Research Center in Bioresources for Agriculture, Industry and Medicine, Chiang Mai University, Chiang Mai 50200, Thailand

**Keywords:** Lanna mural painting, microbial community, biodeterioration, next generation sequencing

## Abstract

**Simple Summary:**

In this study, we compared microbial communities in Lanna mural paintings in temples with different numbers of visitors using culture-dependent and culture independent approaches. The results showed that microorganisms could damage the colors that are used on murals. The process of degradation involved the production of organic acids and formation of the calcium crystal. Furthermore, we found that the site with higher number of visitors is associated with microbial contamination from humans while the site with lower number of visitors had higher saprotroph population. Further research into these microorganisms, their activities and functional roles may provide crucial information to aid the preservation of mural paintings.

**Abstract:**

Lanna painting is a unique type of painting in many temples in the Northern Thai region. Similar to most mural paintings, they usually decay over time partly due to the activity of microbes. This study aimed to investigate the microorganisms from two Lanna masterpiece paintings in two temples that differ in the numbers of visitors using both culture-dependent and -independent approaches. The microorganisms isolated from the murals were also tested for the biodeterioration activities including discoloration, acid production and calcium precipitation. Most microorganisms extracted from the paintings were able to discolor the paints, but only fungi were able to discolor, produce acids and precipitate calcium. The microorganism communities, diversity and functional prediction were also investigated using the culture-independent method. The diversity of microorganisms and functional prediction were different between the two temples. *Gammaproteobacteria* was the predominant group of bacteria in both temples. However, the fungal communities were different between the two temples as *Aspergillus* was the most abundant genus in the site with higher number of visitors [Buak Krok Luang temple (BK)]. Conversely, mural paintings at Tha Kham temple (TK) were dominated by the *Neodevriesia* genera. We noticed that a high number of visitors (Buak Krok Luang) was correlated with microbial contamination from humans while the microbial community at Tha Kham temple had a higher proportion of saprotrophs. These results could be applied to formulate a strategy to mitigate the amount of tourists as well as manage microorganism to slow down the biodeterioration process.

## 1. Introduction

Mural paintings are a form of cultural heritage that is associated with a sense of identity and often exploited to benefit the tourism industry and economy [1]. Management and preservation of murals, especially those with cultural significance such as paintings in churches, require large amount of public funding and therefore are often neglected in developing countries and underdeveloped areas [2]. Such poor management is the case in the north of Thailand where the Lanna Kingdom was founded in the 14th century. Lanna heritage, including architecture and mural paintings, has been on the tentative list for UNESCO heritage sites since 2015, reflecting its uniqueness in culture and history [3,4]. Despite the cultural significance, deterioration of Lanna architecture and murals is still progressing and require urgent care.

Mural paintings in archaeological sites normally decay over time due to abiotic factors, such as humidity, temperature, and biotic factor including microorganisms [5]. Studies have reported that temperature and moisture play a crucial role in controlling the microbial community composition [6,7]. Furthermore, paintings also face biodeterioration caused by biological agents such as plant [8], animals [9] and microorganisms. Microorganisms are important factors in the degradation process of materials including concrete building, mortar and some color paintings [10]. Many studies in the past decade found that fungi and bacteria contribute to the destruction of the painting equipment, ceramics, mummies, and books. These microorganisms can penetrate the materials and cause damage through different pathways such as production of acids and digestive enzymes, or direct physical damage. 

Deterioration of building surfaces through microorganisms is associated with environmental factors including pH, temperature, humidity, nutrient sources, and biomineralization [11,12,13]. For instance, the moist environment in caves and underground tombs is an ideal environment that supports the growth of fungi. Black mold is a type of fungus and one of the most common culprits associated with the destruction of mortar and soft materials. In a less humid, semi-arid and arid environment, cyanobacteria, bacteria, algae and lichens are the primary groups responsible for material corrosion [14]. The microbes’ physical properties, as well as enzymatic biodeterioration, can cause damage and destroy artworks even those produced from modern materials such as polymer [15]. Paintings and murals are normally carried out on organic materials and provide carbon source for fungi and other microorganisms. Studying the bacterial and fungal communities on those paintings will provide an insight into dominant groups of microorganisms that cause biodeterioration and further information on their biodeterioration activities on the material surface and structure [16,17,18].

The impact of tourism on mural painting decay has often been less considered, but studies have shown that tourism is associated with contamination of microorganisms in archaeological sites [2]. Reports have shown that the airflow in more crowded archaeological sites contain higher amount of fungal spores including *Aspergillus* genus, which is commonly found on human feet, and *Malassezia,* normally found on human armpits [2,19,20]. *Aspergillus* spp. are known to produce cellulolytic enzyme and acids that are biodeterioration agents [21]. Many studies have isolated and identified *Aspergillus* and *Curvularia* genera from mural paintings using morphological and molecular methods [22,23,24]. These genera were found to be the dominant fungal genera on the surface of paintings along with the bacteria *Bacillus*.

Microbial communities in environmental samples can be assessed based on various culture-dependent and culture independent approaches or a combination of both approaches [25,26,27] The culture-dependent approach is a primary method employed to investigate the culturable viable microorganism in the environmental sample [27,28]. This conventional method is based on isolation techniques using various media culture types and, depending on microbial physiological requirements, different taxa and communities can be isolated [27,29]. Various media that are used in this method include universal media (such as nutrient agar for bacteria and Potatoes dextrose agar for fungi) as well as specific media, which can be used to targeted different microbial taxonomic and functional groups [27]. Culture-dependent approaches focus on living components of the microbial community and allows researchers to obtain bacterial taxonomic information with the aid from multigene phylogenetic analyses of relevant genes [25,26]. Moreover, these approaches may provide microbial materials that can be further used in subsequent work [30]. However, Daniel (2005) reported that only approximately 1% of environmental bacteria [31] and 30% of fungi are culturable [32], inferring a bias of growing microbial communities from environments. Nonetheless, a study argues that a significant proportion of microbial communities can be isolated depending on selected media, isolation methods and culturing conditions [33]. To date, it is still debatable whether the majority of microbes in environmental samples are indeed culturable [34]. Since microbial communities in environmental samples consist of culturable and non-culturable members, culture independent techniques have been developed to characterize microbial community structure to detect both unculturable and non-culturable microbes in the environments [35]. These approaches are based on genetics and genomics information especially DNA and RNA [36,37]. These approaches are generally less laborious but require more efforts on molecular biology and bioinformatics. Culture-independent approaches have been rapidly developed over the past decade due to the applications of high-throughput sequencing technologies such as Next Generation Sequencing (NGS, e.g., 454 pyrosequencing, Illumina sequencing) and long-read Third Generation Sequencing (TGS, e.g., PacBio, Oxford Nanopore [38,39,40] Based on genetic and bioinformatic information obtained from culture-independent approaches, we can reasonably characterize the diversity and community composition as well as predict potential interactions and functions of microbes in various environments [41,42,43]. However, there are some pitfalls to culture-independent approaches, particularly relating to PCR biases [42]. Thus, various lines of studies suggest that combining the culture-dependent and culture independent approaches can give a sounder conclusion to the total microbial communities in environmental samples [25,26,27]. Therefore, in this study, we investigated microbial communities on Lanna mural painting using both approaches to identify as many practicable microbial communities and also their possible activities in their environments.

Next generation sequencing (NGS) known as high-throughput sequencing, is the catch-all term used to describe several different modern sequencing technologies. NGS is an important method to characterize and describe microbial communities on the mural paintings [15]. NGS was used to study the fungal community from ancient painted sculptures in Majishan Grottoes. The study revealed that *Firmicutes* and *Ascomycota* were the predominant groups of bacteria and fungi, respectively, among the collected samples [44]. *Firmicutes* was also the predominant bacterial group from wall paintings preserved of the Tiantishan Grottoes and ancient wall paintings of the Mogao Grottoes in Chin [45,46], sculptures and paintings in Italy [47], and Leonardo da Vinci’s drawings [48]. These studies have identified various bacteria and fungi, which when combined with the elucidation of functional roles, may provide an insight to the fungi’s position in the ecosystem [2]. Despite multiple studies on microorganism communities on mural paintings in various sites, there is still a lack of the knowledge of microbes in murals in the tropic as most of these studies were performed in temperate areas and also in well-managed sites. This study aimed to identify the microbial communities in mural paintings from the Northern region of Thailand using culture-dependent and culture-independent approaches. Moreover, the impact of mural site visit frequency was conducted to determine the link to biodeterioration. We hypothesized that the microbial community differed between the temples due to many factors, including temperature, humidity, light concentration and the number of visitors. The results could be used as a model for monitoring, preserving and guiding cultural site management in tropical areas and make the tourism industry more sustainable.

## 2. Materials and Methods

### 2.1. Sample Collections 

#### 2.1.1. Physical Properties Measurement and Visiting Frequency Estimation 

**Figure 1:**
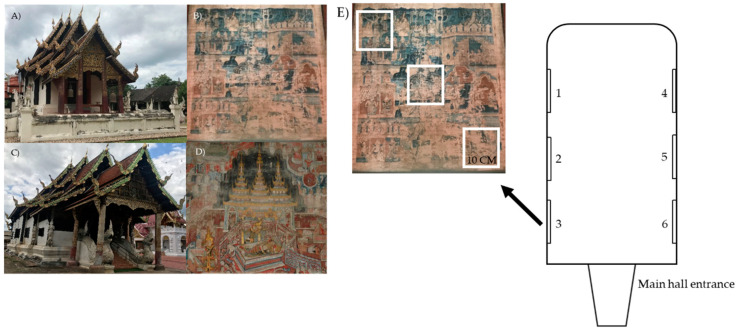
Buak Krok Luang Temple (**A**), mural painting of the Buak Krok Luang Temple (**B**), Tha Kham Temple (**C**), mural painting of the Tha Kham Temple (**D**) and sampling areas within the temples, the white frames demonstrate 3 sampling replications in each area (**E**).

Two temples, Buak Krok Luang Temple (Figure 1A,B) and Tha Kham Temple (Figure 1C,D), Chiang Mai province, Thailand, were chosen to examine the physical properties. Murals in these temples were painted in the 19th century by the same group of artists using Lanna-Shan style and the main colors are red and green. The physical properties, including temperature, relative humidity and light concentration, were recorded. Temperature and relative humidity were measured using temperature and humidity meters (Testo 174-H set, Bangkok, Thailand), while the TM-201 Lux/Fc light meter (TENMARS, Taipei, Taiwan) was used to measure the light concentration. The physical properties were measured in the main hall of the temples during the sample collection period (October, 2019). The visiting frequency estimation was conducted by interviewing the head monk of each temple. 

#### 2.1.2. Microorganism Collection 

In this case, 12 samples were collected from the mural paintings (Figure 1 and Figure S1) (average area of the murals from both temples is 1800 ± 20 cm^2^) at the main hall of both temples. Six samples (3 replicates/sample) were collected from each temple as shown in Figure 1E (The samples were collected from number 1–6 from each temple). To minimize damage on the paintings, samples were randomly collected from various colors by lightly stroking cotton swabs on the painting surface (cotton swab method). The cotton swabs were kept in 0.1% tween 80 and was later used for the culture-dependent method and DNA/RNA Shield (Zymo Research, Irvine, CA, USA) for the culture-independent study.

### 2.2. Culture-Dependent Study 

#### 2.2.1. Isolation and Identification of Microorganism

The microbial samples were serially dilute tenfold by 0.85% (*v*/*v*) NaCl solution to yield the concentrations range of 10^−1^ to 10^−5^. Then, 100µL of each concentration was spread on Potato dextrose agar (PDA) plates for fungi and Tryptic soy agar (TSA) for bacteria. The plates were incubated at 30 °C for 24–72 h. Individual bacterial colonies were then picked from each plate and streaked on a new TSA plate for bacteria. Mycelium from each fungal colony was picked from the PDA plate and placed it on a new PDA plate until they formed a single colony. Genomic DNA of fungal and bacterial isolates from selected colonies were extracted using a rapid extraction method for fungi (Thermolysis method-26) and DNA extraction kits (Invitrogen, Waltham, MA, USA) for bacteria. Each DNA sample was amplified using primer 27F/1492R for bacteria and ITS4/ITS5 for fungi. The total reaction volume was 25 μL and contained 1 μM of amplicon PCR forward and reverse primers (5μL each), 2× KAPA HiFi HotStart ReadyMix (Roche, Basel, Switzerland) and 2.5 μL of the microbial DNA (5ng/μL). The DNA were amplified following the cycling conditions, denature at 95 °C for 3 min followed by 35 cycles of 95 °C for 30 s, 55 °C for 30 s, 72 °C for 30 s, and the final extension at 72 °C for 5 min, then held at 4 °C. PCR for fungal samples were performed using the same PCR steps with the following modifications—the annealing temperature was changed to 55 °C for 40 s and the extension time was changed to 40 s instead of 90 s and held at 4 °C. All PCR products were analyzed with the dideoxy method by Macrogen (Macrogen Inc., Seoul, Korea). All sequences were edited and aligned using Bioedit (version 7.1.0) [49]. These sequences were blasted using the BLASTn tool in the GenBank database and phylogenetic tree construction was performed using MEGA X [50] with kimura-2 model.

#### 2.2.2. Biodeterioration Test of Microorganism and Acid Production Study of Microorganism

Biodeterioration ability of the microorganisms were investigated on 0.8% malachite green and crimson red agar (TSA for bacteria and PDA for fungi). Each mycelium from fungal isolate was spread on PDA plates then incubated for 24 h. The fungal mycelium grown on the PDA plates was picked using a 7 mm cock borer sterilized with 95% ethanol and placed on PDA plate mixed with malachite green or crimson red (Krayarong Thai Mural pigment Studio, Nontaburi, Thailand). Both colors were used as they are paint colors used in both temples [51]. Bacterial biodeterioration was investigated using an agar well diffusion method and TSB as a negative control. Each isolate was incubated in Tryptic soy broth (TSB) for 24 h. The isolates were adjusted to 1.0 McFarland standard and tested for the biodeterioration activity by incubating on TSA mixed with malachite green or crimson red for 24–48 h. After incubation, clear zones and any undesirable changes in color on the plates were determined. Each microbial isolate with any visible sign of biodeterioration was further investigated for their acid production. The isolates were placed in Potato Dextrose Broth (PDB) for fungi and TSB for bacteria and the initial pH was measured. After incubating for 24 and 72 h for bacteria and fungi, respectively, the pH was measured again as the final pH [52,53,54]. The suspensions were centrifuged and filtered into a new tube. All solutions which pH lower than the initial were subject to high performance liquid chromatography (HPLC) to analyze the solutions on Agilent Technology 1260 infinity (Santa Clara, CA, USA), using five acids as standard references including acetic acid, ascorbic acid, malic acid, lactic acid and oxalic acid on a Purospher^®^STAR RP-18 endcapped 150 cm × 4.6 mm, 5 µm column (Merck, Darmstadt, Germany).

#### 2.2.3. Mineralization and Assay

The microbial colonies from the previous step were incubated in B4 medium according to Ma et al. [55]. The calcium crystal morphology was observed under a scanning electron microscope JSM 5910 LV (JEOL, Akishima, Tokyo, Japan) and their chemical composition was analyzed by Energy Dispersive X-ray Spectrometer (EDS) (JEOL, Akishima, Tokyo, Japan).

### 2.3. Culture-Independent Study

#### 2.3.1. Genomic DNA Extraction, Sequencing, Bioinformatics and Data Processing for Fungi and Bacteria

The 12 samples collected from the temples were centrifuged at 10,000 rpm for 15 min. The solution was resuspended using the lysis buffer in the Quick-DNATM Fecal/soil Microbe Microprep kit (Zymo research, Irvine, CA, USA) according to the manufacturer’s protocols. Each DNA sample was amplified using the same conditions shown in Section 2.2.1.

Next Generation Sequencing (NGS) of each sample collected from the temples was performed within the V3-V4 regions using 341F (-CCTACGGGNGGCWGCAG-) and 805R (-GACTACHVGGGTATCTAATCC-) primers for bacteria and ITS1-ITS2 regions using ITS1F (-CTTGGTCATTTAGAGGAAGTAA-) and ITS2R (-GCTGCGTTCTTCATCGATGC-) primers for fungi. Amplicon sequencing was performed using Illumina (MiSeq) Macrogen (Macrogen Inc., Seoul, Korea). The raw sequences were analyzed on QIIME2 software version 2020.11 [56]. Singleton and chimeric sequences from the raw sequences data were removed before the analysis of taxonomic classification.

All sequences were trimmed using their forward and reverse primer (341F and 805R for bacterial and ITS1F and ITS2R for fungi) in QIIME2 version 2020.11. The sequences were denoised with DADA2 [57] to remove low quality reads and merge high quality sequences together. Rarefaction curve and data normalization parameters were set depending on individual data values. All bacterial sequences were classified using Greengene database version 13.8 and Unite version 8.2 was used to classify fungal sequences [58,59]. All microorganism sequences were deposited in the GenBank database (MZ569607-MZ569623 for fungi and MZ577077-MZ577085 for bacteria). In this case, 22 raw data sequences obtained from Illummina (MiSeq) were deposited into the bioproject number PRJNA746729.

#### 2.3.2. Microbial Diversity and Function Study

Stacked bar plots of the bacterial and fungal communities were constructed using the “ggplot2” package in R software version 4.1.1 [60]. The data were categorized into three files including amplicon sequence variant (ASVs) file, taxa file and sample data file, which were then used to analyze the alpha- and beta-diversity using the “phyloseq” and “ggplot2” packages in the R software version 4.1.1. The alpha-diversity was analyzed using three indexes including simpson, chao1 and shannon index. The Non-metric Multi-dimensional Scaling (NMDS) was calculated based on Bray-Curtis indexes using the “phyloseq” package in R-studio.

QIIME2 was used to predict functions. PICRUSt2 software [61] was used to predict the bacterial and fungal metagenome functions based on the marker genes. Bacterial and fungal ASVs data were imported and performed the prediction using picrust2_pipeline.py from https://github.com (Accessed date: 10 June 2021). Microorganism functional prediction results were used to construct a heat map using R-studio with “gplots” and “ggplot2” packages.

#### 2.3.3. Statistics Analysis

The significant level for all diversity analyses were set at *p* < 0.05. Each statistical analysis was carried out using R software version 4.1.1. The microbial community significant difference for both temples were calculated using R software version 4.1.1 with an unpaired *t*-test with Welch’s correction.

## 3. Results

### 3.1. Physical Property Measurements

The temperature, relative humidity and light concentration were recorded in the main hall of Buak Krok Luang and Tha Kham temples. The temperature at Buak Krok Luang temple was higher than that at the Tha Kham temple (31.3 ± 0.4 °C and 28.7 ± 0.5 °C, respectively, *p*-values = 3.8 × 10^−6^ °C). The light concentration of Buak Krok Luang temple was also higher (11.4 ± 0.3 and 7.9 ± 0.4 Lux, respectively, *p* = 7.29 × 10^−8^). The relative humidity of Buak Krok Luang temple was lower than that of Tha Kham temple (53.4 ± 1% and 68.8 ± 0.9%, respectively, *p* = 2.07 × 10^−10^). The estimated number of visitors per month at Buak Krok Luang temple was higher than that at the Tha kham temple (400 and 160, respectively).

### 3.2. Microorganism Isolates and Their Bioterioration Activity

**Figure 2:**
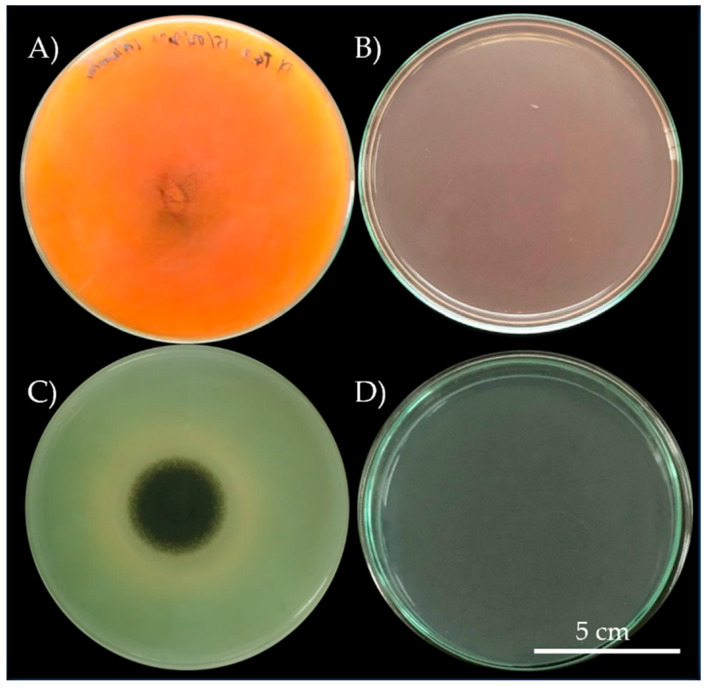
Biodeterioration activity of isolate *Aspergillus aculeatinus* (**A**) on crimson red and isolate *Aspergillus piperis* (**C**) on malachite green, compare with crimson red control (**B**) and malachite green control (**D**).

In total, we obtained 9 pure bacterial cultures from mural paintings (5 isolates from Buak Krok Luang Temple and 4 isolates from Tha Kham Temple). In addition, we obtained 17 fungal pure cultures, 9 from Tha Kham Temple, 6 from Buak Krok Luang Temple and 2 from both locations. All fungal and bacterial colonies were tested for their biodeterioration ability on PDA for fungi and TSA for bacteria, these agars were mixed with crimson red and malachite green colors (0.8%. w/v). Only 3 (33.33%; 3/9) isolates of bacteria (BK3, BK4 and TK3) showed the biodeterioration ability on TSA mixed with malachite green, indicated by brown circles around the colonies on the agar after incubation. No change was observed on TSA mixed with crimson red Table S1. On the other hand, 11 (64.7%; 11/17) fungal colonies showed the biodeterioration activity on PDA plates mixed with the colors Table S1. Colonies BK1 and TK6 (11.76%; 2/17) caused the changes in the color tone on both malachite green and crimson red while colonies TK2, TK3, TK4, TK5, BK2, BK3 and BTK2 (41.17%; 7/17) caused the changing only on crimson red. Only 2 colonies (BTK1 and TK8; 11.76; 2/17) caused the color change on malachite green plates, resulting in the structural change in colors on PDA as evident by the hue change of crimson red to orange and malachite green to olive (Figure 2A,C).

### 3.3. Organic Acid Production and Calcium Crystal Formation of Microorganism Isolates

**Figure 3:**
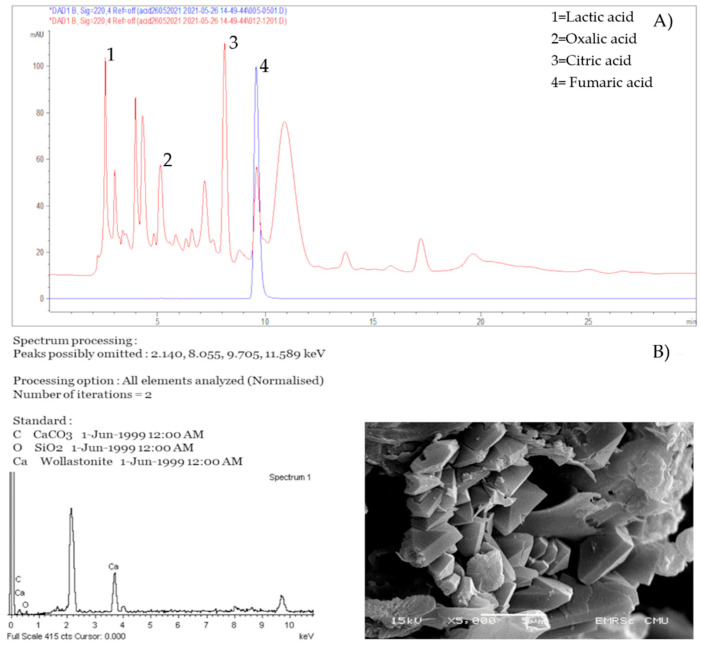
Acid production of *Aspergillus fumigatus* (red peak) compared with fumaric acid standard (blue peak) (**A**) and Calcium formation and EDS analysis of *Aspergillus piperis* (**B**).

Isolates with the biodeterioration ability were tested for the ability to produce acid. The isolates were incubated in tubes filled with PDB for fungi and TSB for bacteria and pH was measured Table S2. Then, they were subject to HPLC. The HPLC results showed that all fungi with the biodeterioration ability could produce organic acids including lactic, oxalic, malic, fumaric, citric and acetic acids. These acids are normally secreted from fungi that are found on mural paintings [62,63]. Most of these fungi produced citric acid and lactic acid (52.9%; 9/17), 5 produced malic acid and succinic acid (29.4%; 5/17), and 6 produced fumaric acid. Only a few of the fungi produced acetic acid (11.7%; 2/17) and oxalic acid (17.6%; 3/17) Table 1 and Figure 3.

As the ability to form calcium is indicative of the ability to cause staining on and damage paintings [64,65,66,67], all fungal isolates from both temples were also tested for their calcium formation using scanning electron microscope. None of bacterial isolates could precipitate calcium carbonate. On the other hand, 2 fungal isolates could form calcium crystal on B4 agar Table 1. The isolates were identified to be *A. niger* and *A. piperis*. Both fungal isolates had the same calcium oxalate crystal structure as shown in Figure 3B.

### 3.4. Microbial Community Structure in Mural Paintings Characterized by Culture-Independent Molecular Technique

**Figure 4:**
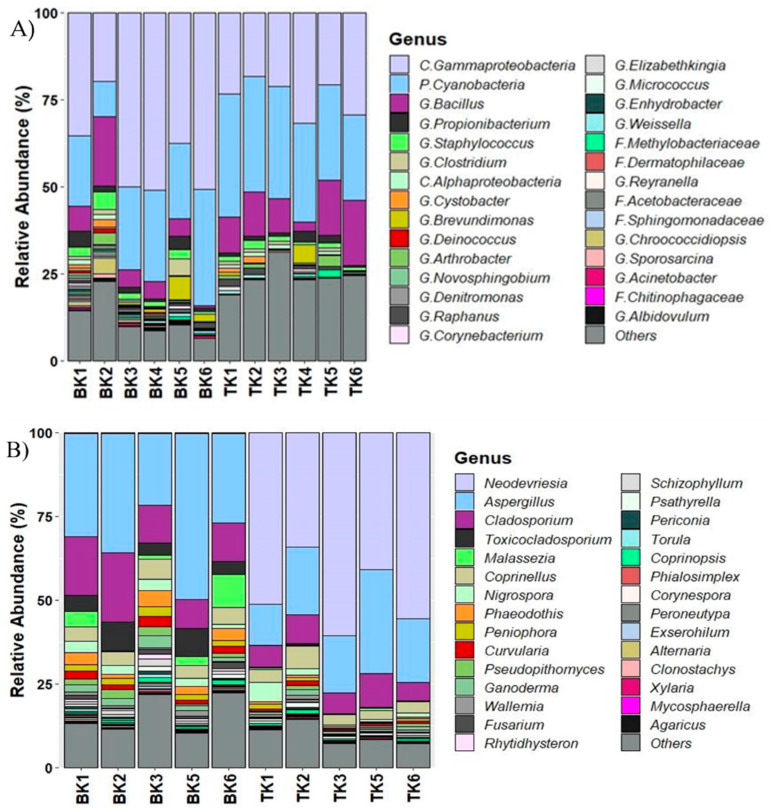
Microbial community composition of bacteria (**A**) and fungi (**B**) from two mural paintings from Buak Krok Luang temple (BK) and Tha Kham temple (TK). The stacked bar-plots were derived from high-throughput sequencing data.

A total of 4760 ASVs were obtained from bacterial sequences. The data were edited to superficially-identified ASVs assembly and deeper-identified groups, out of which 29 ASVs were used to analyze the bacterial communities. *Gammaproteobacteria* was the dominant group (>30%, *p* = 0.005) among the samples from Buak Krok Luang temple and about 25% of the samples from Tha Kham temple, followed by Cyanobacteria (>20%, *p* = 0.01) and *Bacillus* (10%, *p* = 0.06). The fungal community was analyzed based on a total of 4755 ASVs and the data were edited to 31 ASVs. *Aspergillus* was the most abundant group (>25%) in Buak Krok Luang temple followed by *Cladosporium* (15%, *p* = 0.03). On the other hand, *Neodevriesia* was the dominant group (>45%) followed by *Aspergillus* (>15%) in Tha Kham temple. (Figure 4A,B).

### 3.5. Comparison of Microbial Diversity and Community Composition Using NGS

**Figure 5:**
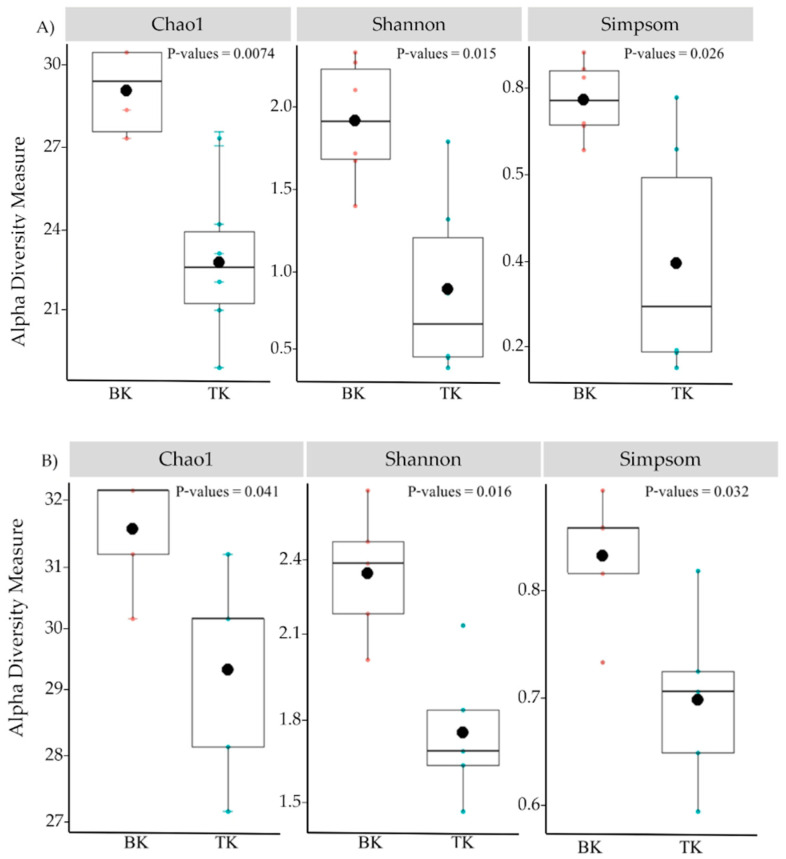
Alpha diversity of bacteria (**A**) and Fungi (**B**) of mural paintings according to Chao1, Shannon and Simpson indices in samples collected from Buak Krok Luang Temple (BK) and Tha Kham Temple (TK), black dots and error bar represent the means and Standard deviation of each barplot.

The microbial diversity was analyzed using the results from high-throughput sequencing of microorganism samples. The total bacterial reads after trimming the pair end was 1,190,813 and further reduced to 805,013 sequences after denoising and cutting chimeric sequences. The total fungal reads were 1,131,221 and reduced to 717,291 reads after denoising and cutting chimeric sequences. All sequences from both temples were clustered into 4755 and 4777 ASVs for bacteria. Alpha diversity was calculated using three indexes, including Choa1, Simpson and Shannon. The results showed that the samples from Buak Krok Luang were higher in all indexes than samples from Tha Kham temple for both bacteria and fungi (Figure 5A,B).

**Figure 6:**
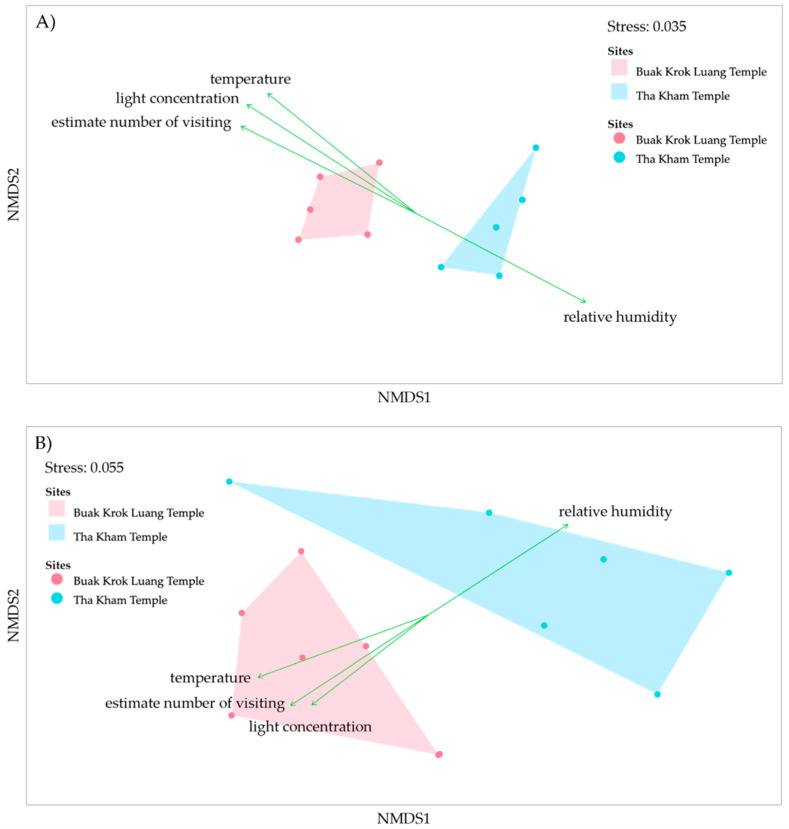
Non-metric Multi-dimensional Scaling (NMDS) with environment parameters of microorganism, bacteria (**A**) and fungi (**B**) on mural paintings from the two temples. The red dots represent the samples from Buak Krok Luang temple while the blue dots represent the samples from Tha Kham temple. The green arrows indicate the factors which impact on the microorganism community in each temple.

The non-metric multidimensional scaling (NMDS) was calculated based on Bray-Curtis indexes to analyze the difference between microorganism communities in the two locations. The result illustrates that the bacterial and fungal community compositions between the two temples were distinguished from each other (Figure 6A,B). The stress of the bacterial and fungal NMDS, indicative of deviation, is 0.035 for bacteria and 0.055 for fungi. The data collected for each temple including estimated number of visitors, relative humidity (RH), light concentration and temperature were used to determine the microorganism diversity. The results showed that the environmental factors were the dominant factors that caused the differences in the microorganism diversity.

### 3.6. Functional Roles of Microorganisms Detected from the Mural Paintings

The bacterial functional prediction heat map shows that aerobic respiration I (cytochrome C) was the most abundant function found in bacteria from Buak Krok Luang temple. On the other hand, there were many prominent functions in the samples collected from Tha Kham temple including GDP-mannose biosynthesis, fatty acid and beta-oxidation I and L-isoleucine biosynthesis II (Figure S2). The heat map of the fungal functional pathway prediction showed a similar pattern in that two aerobic respiration pathway were the most abundant in Buak Krok Luang temple, while fatty acid and beta-oxidation were less abundance in Tha Kham temple. (Figure S3).

### 3.7. Microbial Identification

All bacterial and fungal isolates were identified using the 16S RNA gene for bacteria and the ITS region for fungi. Genomic DNA of bacterial colonies with the biodeterioration ability were extracted and sequenced by dideoxy sequencing. The sequences were compared to those in the NCBI database, and the bacterial sequences were used to construct a phylogenetic tree to explore their evolutionary relationship. The phylogenetic tree categorized 9 cultured bacteria isolates into 9 different species (Figure S4). The dominant bacterial group was *Staphylococcus* genus (67%), and 11% of *Bacillus, Klebsiella* and *Enterococcus*. Genomic DNA of fungi isolates with the biodeterioration activity were also extracted and sequenced using the same method as the bacteria isolates. *A phylogenetic* tree was constructed based on the sequences of the ITS region. The sequences were categorized into 17 different species including, *Penicillium citrinum*, *P. hetheringtonii*, *P. oxalicum*, *P. polonicum*, *Aspergillus fumigatus*, *A. aculeatinus*, *A. flavus*, *A. niger*, *A. piperis*, *Trichoderma longibrachiatum*, *T aethiopicum*, *T. harzianum*, *Fusarium solani*, *F. equiseti*, *F. proliferatum*, *Curvularia nodosa* and *Coprinellus radians*. *Rhizopus* species were used as an outgroup in the phylogenetic tree Figure S5.

### 3.8. Comparison between High-Throughput Sequencing and the Culture-Dependent Method

**Figure 7:**
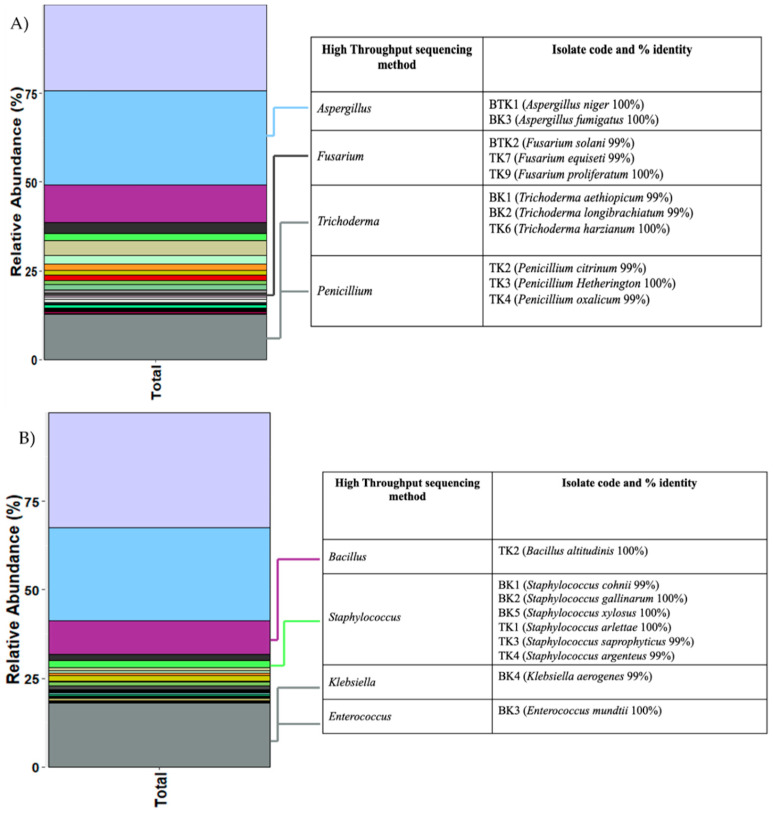
Ratios of fungal (**A**) and bacterial (**B**) communities based on high-throughput sequencing method illustrated in stacked-barplot. Details of each isolate are shown in the table on the right (TK; Tha Kham temple, BK; Buak Krok Luang temple).

Sequences of fungal and bacterial isolates from the culture-dependent method were compared to the data from the high-throughput sequencing method. This enabled us to reasonably identify the fungi and bacteria from Illumina sequencing. The fungal taxa from Illumina (MiSeq) sequencing were matched with the isolates including *Aspergillus*, *Trichoderma*, *Fusarium* and *Penicillium*. *Aspergillus* was the dominant taxa in the fungal community. However, they made up of only 11% (2/17) based on the results from the culture dependent method. *Fusarium* was found at 1% based on the high-throughput method but at 17.6% based on the culture-dependent method. *Trichoderma* and *Penicillium* made up <1% of the fungal community (Figure 7A). Both methods showed that *Staphylococcus* sp. and *Bacillus* sp. were the dominant groups. *Klebsiella* and *Enterococcus* sp. were less abundant based on high-throughput sequencing, but they were found more frequently in the culture-dependent method. (Figure 7B)

## 4. Discussion

Abiotic and biotic factors such as bacteria, temperature, and humidity cause mural paintings on archeological sites to degrade over time. Studying these factors may provide an insight into the deterioration process of mural paintings. In this study, we found that physical property measurements of both temples were distinct. The average temperature and light concentration at Tha Kham temple (a rural area close to a national park) were lower than those at Buak Krok Luanng temple (a city area). Moreover, the relative humidity at the Tha Kham temple was about 30% greater than that at the Buak Krok Luang temple. The number of visitors at both temples was also different in that Tha Kham Temple opens the main hall only on Buddhist days (4 times a month), but Buak Krok Loung temple is a famous tourist site where visitors come to visit the mural paintings every day. These differences in physical factors may influence the distinct microorganism communities and result in different rates of biodeterioration activity. Therefore, a mural conservation method may have to be tailored to each temple depending on the diversity of microorganisms, which can possibly be predicted based on the physical environment.

The biodeterioration activity results showed that most fungi from the temples could cause a change in color on crimson red, while some showed the activity on malachite green. Interestingly, the bacterial biodeterioration results revealed that none of the bacterial isolates were able to change the color structure of crimson red but some were able to alter the color of malachite green. The ability of microorganisms to break down one or both colors may be dependent on the chemical structure of each color, which governs the physical and chemical properties [68]. 

The investigation of fungal acid production was performed using HPLC to detect organic acids. This study showed that, *T. aethiopicum, T. longibrachiatum, T. harzianum* and *F. solani* could produce organic acids in vitro. Lines of evidence have shown that many species in *Aspergillus* and *Penicillium* genera have the ability to produce a large amount of organic acids [62,63]. The production of acids can promote fungal proliferation and fade the painted colors, which further causes damage to mural painting materials and subsequently deterioration [69,70,71]. The ability to form calcium oxalate of the *Aspergillus* genera on agar was also reported [72]. In this study only two out of 17 fungal isolates were able to precipitate calcium crystals on a solid agar. The production of oxalate minerals was aided by fungal growth and metabolism, resulting in mechanical changes such as materials cracking as well as cosmetic (pigment discoloration) damage to the painted layer [64,65,66,67].

In this study, we used both culture-dependent and culture independent approaches to analyze the microbial communities associated with the mural paintings. A drawback from the culture-dependent approach was that we only used a rich medium for the isolation (TSA and PDA for bacteria and fungi, respectively). In habitats with actively diverse and high microbial biomass (i.e., soil, rhizosphere, decomposing litter), such nutrient rich media can promote fast-growing microorganisms, which eventually overgrow slow-growing ones, and thus, microbial diversity can be underestimated. However, we obtained low to moderate numbers of bacterial and fungal colonies (7.1 × 10^3^ ± 0.25 and 1.6 × 10^4^ ± 0.3 CFU for bacteria and fungi, respectively) even with such nutrient-rich media. Future study should also include media with similar properties to the environment of the mural painting. To partly overcome this drawback, we compared bacterial and fungal taxa that were detected in high-throughput sequencing but were not detected in our culture-dependent method. Then, we analyzed their potential to degrade the painting (color degrading, acid production and some enzyme activities) using available publications and presented in Table S3. The results showed that Cyanobacteria are commonly detected on the surfaces of materials such as concrete, limestone, and mural painting, and their growth can produce a wide range of patina colors on the material’s surface [16,73,74,75,76,77]. In addition, acids were produced as metabolic byproducts from cyanobacteria and result in damage to the surface of the materials [75]. *Arthrobacter* genera were also found on material surfaces and mural paintings, and their biofilm formation can overlay the painting surface, leading to rosy discoloration of the mural painting [78,79,80]. Moreover, *Cladosporium* genera, which can be isolated from the mural paintings, have been reported to grow over the painting surface and cause harm to the surface layer of the painting (Table S3) [10,24,81,82,83]. Many factors contribute to the biodeterioration capacity of microorganisms including pigment formation, enzymatic secretion and organic acid excretion. Moreover, some fungi are able to dissolve calcium, causing physical damage to mural paintings [84,85,86]. According to Albertano and Urz (1999), microfungi colonizing marble and limestone monuments employ phototrophic microorganisms’ nutrition to synthesize organic acids, which dissolve CaCO_3_ from the substrate [87]. In addition, fungi can penetrate rock material by hyphal growth and biocorrosive activity, which is driven by the excretion of organic acids or the oxidation of mineral-forming cations, especially iron and manganese. Discoloration of the surface due to the excretion of melanin by dematiaceous fungi is also one of their degradation activities [12]. Bacteria also produce some acids and secondary metabolites that cause damage to mural paintings [88]. Furthermore, they can reduce sulfur and ammonium in the material, lowering the strength of mural paintings by erosion of the calcium carbonate to calcium sulfate [89].

Culture-independent studies suggested that microorganisms play a crucial role in the deterioration process on mural paintings and other materials. Physical features of microorganisms, as well as enzymatic biodeterioration and acids production can damage and destroy mural paintings. [15,90,91,92]. Investigation of the microbial community on the mural paintings from two different temples in Chiang Mai province using Illumina sequencing (MiSeq) showed that the fungal community and the relative abundance of fungi at Tha Kham and Buak Krok Luang temples were different. This difference was likely due to the number and frequency of visitors as Buak Krok Lunag had significantly more tourist visits. *Aspergillus* (32.7%) was the dominant taxa on the mural painting from Buak Krok Luang temple, followed by *Cladosporium* (13.9%) while, *Neodevriesia* (48.5%) and *Aspergillus* (19.4%) were the dominant taxa in the Tha Kham temple. Studies have reported that *Aspergillus* and *Malassezia* can be found on human feet. In addition, *Toxicocladosporium* (*Capnodiales*) is an associated with a variety of human skin diseases [93] and therefore these microorganisms were likely contaminants from visitors who visited Buak Krok Luang temple [2,19]. To the best of our knowledge, this is the first study report that *Neodevriesia* (known as Cladosporium-liked) was detected on mural painting. This genus may have contaminated the temple’s environment due to its presence in plants and rock habitats [94]. However, information on functions and environment of *Neodevrisia* is still scarce, while *Cladosporium* has been reported to be an environmental saprophyte in various conditions [95].

The bacterial communities from the two temples were somewhat similar as the result revealed that *Gammaproteobacteria*, Cyanobacteria and *Firmicute* (*Bacillus*) were the dominant taxa on the mural paintings in both temples. However, the relative abundance of the *Gammaproteobacteria* on the paintings from Tha Kham temple was lower than those from Buak Krok Luang temple. The relative abundance of Cyanobacteria at Tha Kham temple was slightly higher than that at the Buak Krok Luang temple and this was likely due the higher humidity at Tha Kham temple, which may contribute to the propagation of Cyanobacteria [96]. It was previously reported that *Gammaproteobacteria* and *Firmucutes* were the main bacterial species detected on paintings [97,98]. Cyanobacteria were also found on the ancient wall paintings of the Mogao Grottoes [26] and in many sites with high humidity similar to the temples and caves [99]. In addition, some species in the *Gammaproteobacteria* class are also known as human pathogens contaminated in the human breath [20] while *Firmicutes* was reported as one of the dominant phyla of the human skin microbiome [100,101].

Studying functional roles of microorganisms and their communities may provide further insight into their roles in the environment. The relative abundance of the bacterial functions from the two temples was very different, but the functional prediction of both bacteria and fungi revealed that aerobic respiratory was the dominant function in samples from Buak Krok Luang’s mural paintings while groups of biosynthesis and fatty acid and β-oxidation were the dominant functions in samples from Tha Kham temple. The fungal functional prediction heat map showed the same pattern as the bacterial functional prediction heat map in that the aerobic respiration pathway was more abundant in Buak Krok Luang temple while fatty acid and β-oxidation was slightly higher in Tha Kham temple. Lanna mural paintings and wall materials were built using many ingredients such as sand, limestone, buffalo skin boiled water, and rubber oil, and these materials were likely to affect the community and functions of bacterial on mural paintings. β-oxidation plays an important function in the degradation of fatty acids from a carbon source in microbes for their growth and proliferation [102] while, in many bacteria, GDP-mannose and mannose are found in cell envelope polymers such as mannolipids, phosphoinositol mannosides (PIMs), and glycoproteins [103,104]. The aerobic respiration function of the microorganism community in the Buak Krok Luang temple was found in many microorganisms including pathogenic microbes when the oxygen concentration is more than 5 mbar [105].

**Figure 8:**
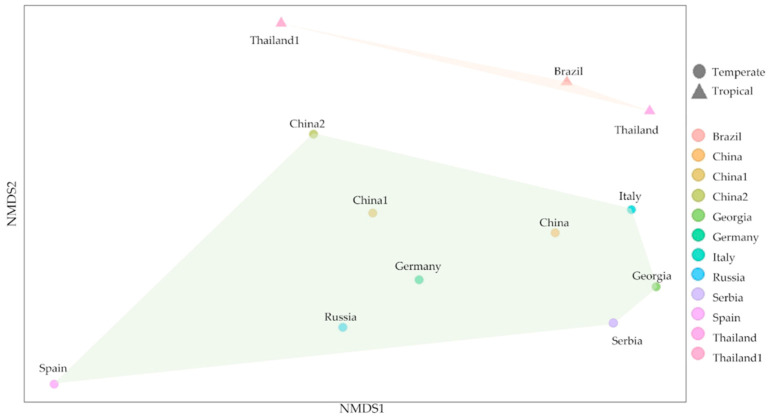
Microorganism diversity comparison among different countries. The triangles are communities from tropical countries (Thailand and Brazil), while circles are communities from temperate countries (China, Georgia, Germany, Italy, Russia, Serbia and Spain).

The culture-dependent method, combined with the culture-independent method, was used to study the microorganism and their biodeterioration abilities. These methods allowed us to visualize the biodeterioration property and identify abundant taxa. Fungal genera, including *Penicilium* sp., *Fusarium* sp., *Aspergillus* sp., and *Curvularia* sp. were found in temples in the central and western part of Thailand [22] and in Brazilian contemporary painting, and 19th-century arts collection [106,107]. Bacterial isolates found in the temples consisted of 6 *Staphylococcus* genus including *S. cohnii*, *S. gallinarum*, *S. xylosus*, *S. arlettae, S. argenteus,* and *S. saprophyticus* and other isolates, *Klebsiella aerogenes*, *Bacillus altitudinis,* and *Enterococcus mundtii*. *Staphylococcus* sp. are normally found in the environment [108]. Moreover, previous studies have reported that *Staphylococcus* and *Bacillus* genera were detected in 17th century paintings [109]. Both of these genera have been associated with biodeterioration and recognized as potential contaminants of artworks [110,111]. Comparison of microorganisms among countries also gives further information on their habitat and diversity as microorganisms from the countries in the same climate tend to group together. Physical factors, including temperature and humidity, are crucial reasons why microorganisms are found together [112,113,114,115]. (Figure 8 and Table S4).

The agreement between the results from high-throughput sequencing and conventional methods enabled us to reasonably identify fungi and bacteria. The metabolic activity of the conventional study revealed that fungi in the *Trichocomaceae* family including *Aspergillus* and *Penicillium* were the dominant groups in the fungal communities in the samples. These families also cause biodeterioration in mural paintings possibly through the production of organic acids and some calcium oxalate crystal. In addition, *Staphylococcus* and some *Gammaproteobacteria*, which were relatively abundant in the bacterial community, also induced biodeterioration. *Staphylococcus* sp. showed the biodeterioration activity on the malachite green. The results suggest that the dominant microorganisms in both temples play an important role in biodeterioration of mural paintings in the temples.

## 5. Conclusions

Microorganism communities in both temples are affected by many factors, including light, temperature, humidity and particularly tourism. Due to the difference in locations, Buak Krok Luang temple, situated in an urban area, had significantly more visits. Contamination of the microorganisms at Buak Krok Luang temple from tourists was higher and they also contribute to biodeterioration in addition to local microorganisms on the painting. On the other hand, microorganism contamination from human was lower in Tha Kham temple. Consequently, local microorganisms were the main factor that contributed to biodeterioration in Tha Kham temple. To conserve mural paintings in the Buak Krok Luang temple, a limit on the number of tourists should be put in place as a main strategy to slow down deterioration, while the strategy to reduce biodeterioration at Tha Kham temple should revolve around decreasing local saprophytic microorganisms.

## Figures and Tables

**Table 1:** Organic acids production and calcium formation of fungal isolates (dark gray indicate positive and light gray indicate negative detection).

Isolates	Code	Acid Production							Calcium Formation
		Malic	Acetic	Citric	Lactic	Fumaric	Succinic	Oxalic	
*Trichoderma aethiopicum*	BK1								
*Trichoderma longibrachiatum*	BK2								
*Aspergillus niger*	BTK1								
*Fusarium solani*	BTK2								
*Aspergillus fumigatus*	BK3								
*Penicillium citrinum*	TK2								
*Penicillium hetheringtonii*	TK3								
*Penicillium oxalicum*	TK4								
*Aspergillus aculeatinus*	TK5								
*Trichoderma harzianum*	TK6								
*Aspergillus piperis*	TK8								

## Data Availability

Publicly available datasets were analyzed in this study. These data can be found under BioProject accession number: PRJNA746729 and accession number MZ569607–MZ569623 for fungal isolation and MZ577077–MZ577085 for bacterial isolation.

## Supplementary Materials

The following are available online at https://www.mdpi.com/article/10.3390/biology11020228/s1, Figure S1: Background level of deterioration on mural paintings from Tha Kham temple (A,B) and Buak Krok Luang temple (C,D), Figure S2: fungal function analysis heat map of samples from Buak Krok Luang and Tha Kham temples, Figure S3: Bacterial function analysis heat map from Buak Krok Luang and Tha Kham temples, Figure S4: Fungal phylogenetic tree was constructed from sequences from the culturable fungi using MEGA-X with kimura 2 model, the bootstrap values were shown on each branch, Figure S5: Bacterial phylogenetic tree was constructed from sequences from the culturable bacteria using MEGA-X with kimura 2 model, the bootstrap values were shown on each branch, Table S1: Bacterial biodeterioration activity on TSA mixed with crimson red (CR) and malachite green (MG) and fungal biodeterioration activity on PDA mixed with the same color, Table S2: pH measurement of microorganism isolates, all of bacterial isolates show slightly increased pH after incubation period while all of the fungal isolates decrease more than 25% based on the initial pH, Table S3: Comparison between biodeterioration activities from previously studies and microorganisms from culture-independent method from this study, Table S4: Basic data relates to Figure 8. The table represents the microorganisms which are found in each country (dark gray indicates that this study did not focus on the organism).
