# Supplementary material for: Identification of Microorganisms Dwelling on the 19th Century Lanna Mural Paintings from Northern Thailand Using Culture-Dependent and -Independent Approaches
Source: Biology (Basel). 2022 Jan 31;11(2):228. doi: 10.3390/biology11020228 (PMC8869426; doi:10.3390/biology11020228)
Supplement: Supplementary file 1 [file biology-11-00228-s001.zip › biology-1490767-supplementary.pdf]

Supplementary Materials

Identification of microorganisms dwelling on the 19th Century Lanna mural paintings from Northern Thailand using culture-dependent and -independent approaches.

Nattaphon Suphaphimol[1], Nakarin Suwannarach[2], Witoon Purahong[3,*], Churdsak Jaikang[4], Kamonpan Pengpat[5], Natthawat Semakul[6], Saranphong Yimklan[6], Surachai Jongjitngam[7], Saiklang Jindasu[8], Sathaporn Thiangtham[8], Panuwan Chantawannakul[1] and Terd Disayathanoowat[1,2,9,*]

1 Department of Biology, Faculty of Science, Chiang Mai University, Chiang Mai 50200 and Thailand; nattaphon.supp@gmail.com; panuwan.c@cmu.ac.th ; terd.dis@cmu.ac.th

2 Research Center of Microbial Diversity and Sustainable Utilization, Chiang Mai University, Chiang Mai 50200, Thailand; suwan.462@gmail.com

3 Department of Soil Ecology, UFZ-Helmholtz Centre for Environmental Research, 06120 Halle (Saale), Germany; witoon.purahong@ufz.de

4 Toxicology section, Department of Forensic Medicine, Faculty of Medicine, Chiang Mai University, Chiang Mai 50200, Thailand; churdsak.j@cmu.ac.th

5 Department of Physics and Materials Science, Faculty of Science, Chiang Mai University, Chiang Mai 50200, Thailand; kamonpan.p@cmu.ac.th

6 Department of Chemistry, Faculty of Science, Chiang Mai University, Chiang Mai 50200, Thailand; natthawat.semakul@cmu.ac.th; Saranphong.Yimklan@cmu.ac.th

7 Department of Thai art, Faculty of Fine arts, Chiang Mai university, Chiang Mai 50200, Thailand; surachai.j@cmu.ac.th

8 The 7th Regional Office of Fine arts, Department of Fine art, Ministry of Culture, Chiang Mai 50300 , Thailand; klangjindasu@gmail.com; thanw3@yahoo.com

9 Research Center in Bioresources for Agriculture, Industry and Medicine, Chiang Mai University, Chiang Mai 50200, Thailand

* Correspondence: terd.dis@cmu.ac.th (T.D.); witoon.purahong@ufz.de (W.P.)

Figure S1. Background level of the deterioration on mural paintings from Tha Kham temple (A,B) and Buak Krok Luang temple (C,D).

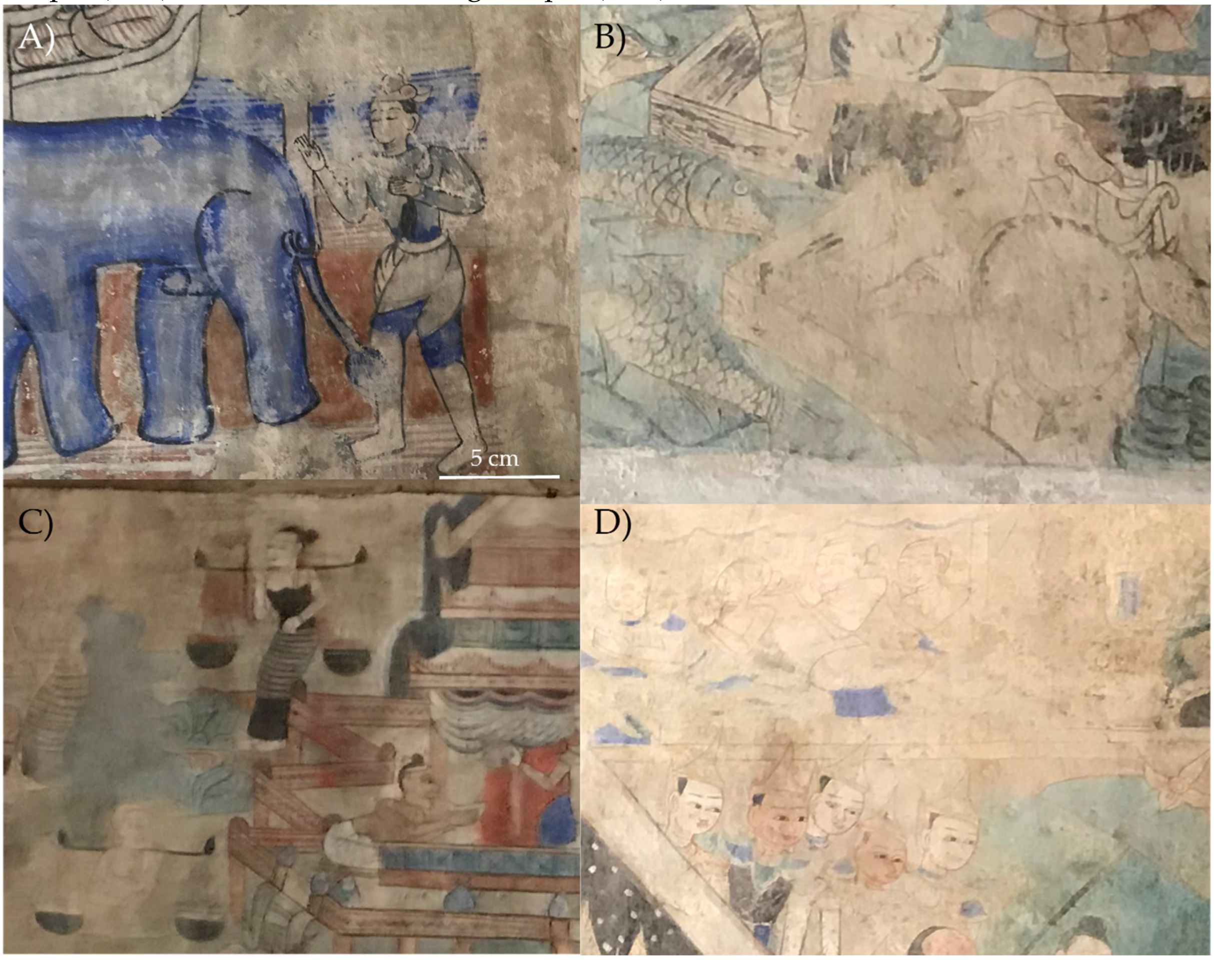

Figure S2. Bacterial function analysis heat map of samples from Buak Krok Luang and Tha Kham temples. The functional pathways are list on the right side of the heatmap.

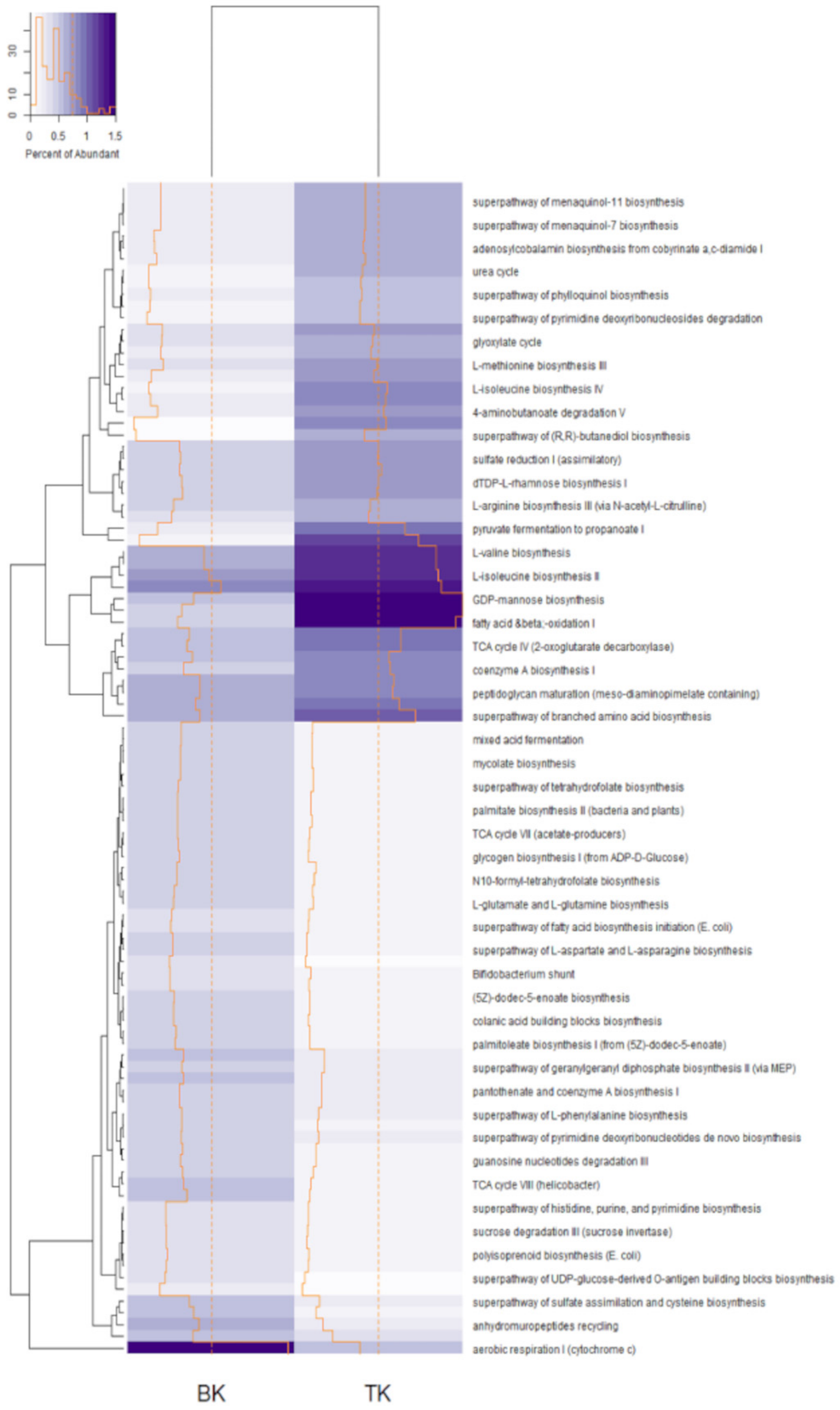

Figure S3. Fungal function analysis heat map from Buak Krok Luang and Tha Kham temples. The functional pathways are listed on the right side of the heat map.

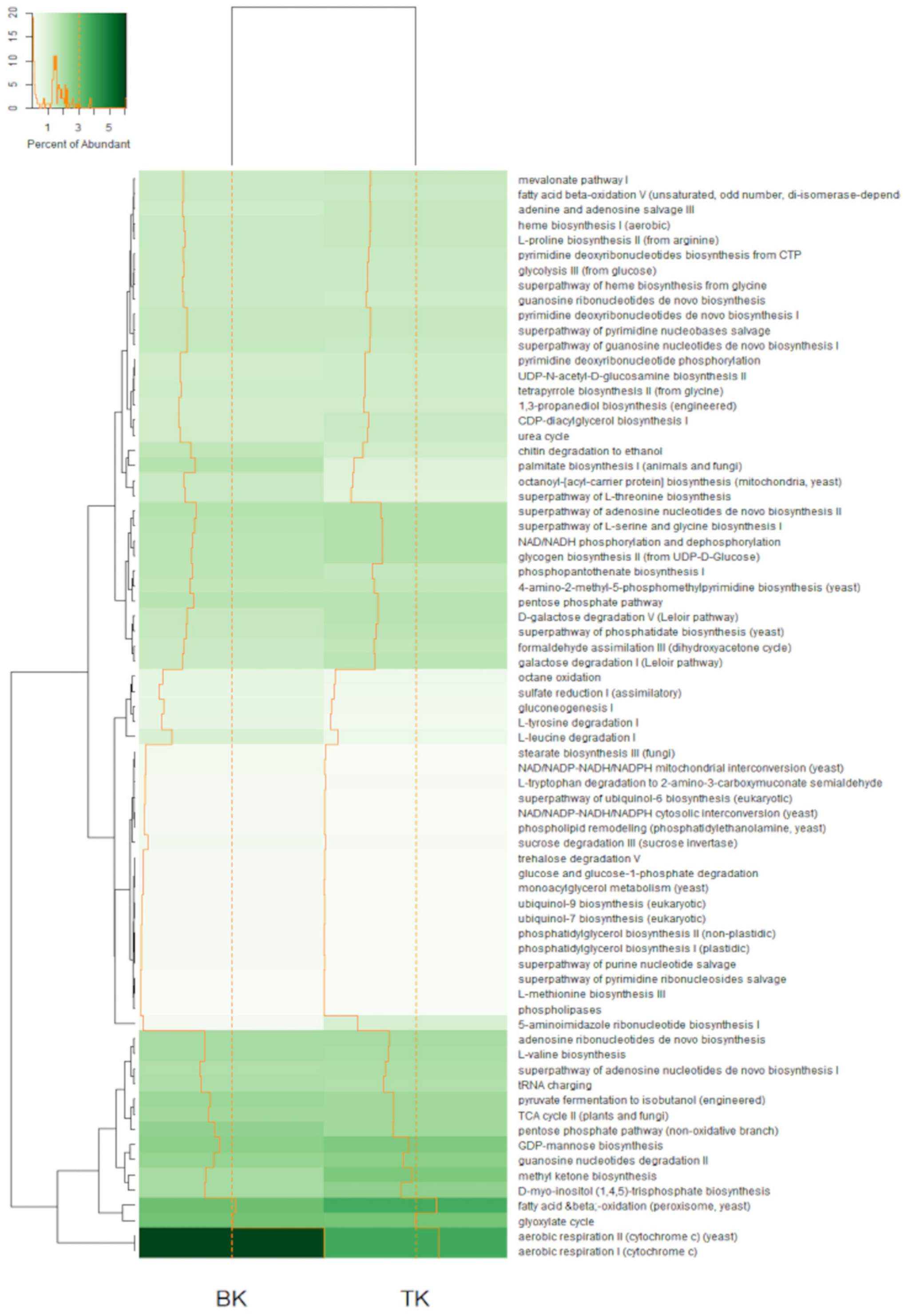

Figure S4. Bacterial phylogenetic tree was constructed from sequences from the culturable bacteria using MEGA-X with kimura 2 model, the bootstrap values were shown on each branch.

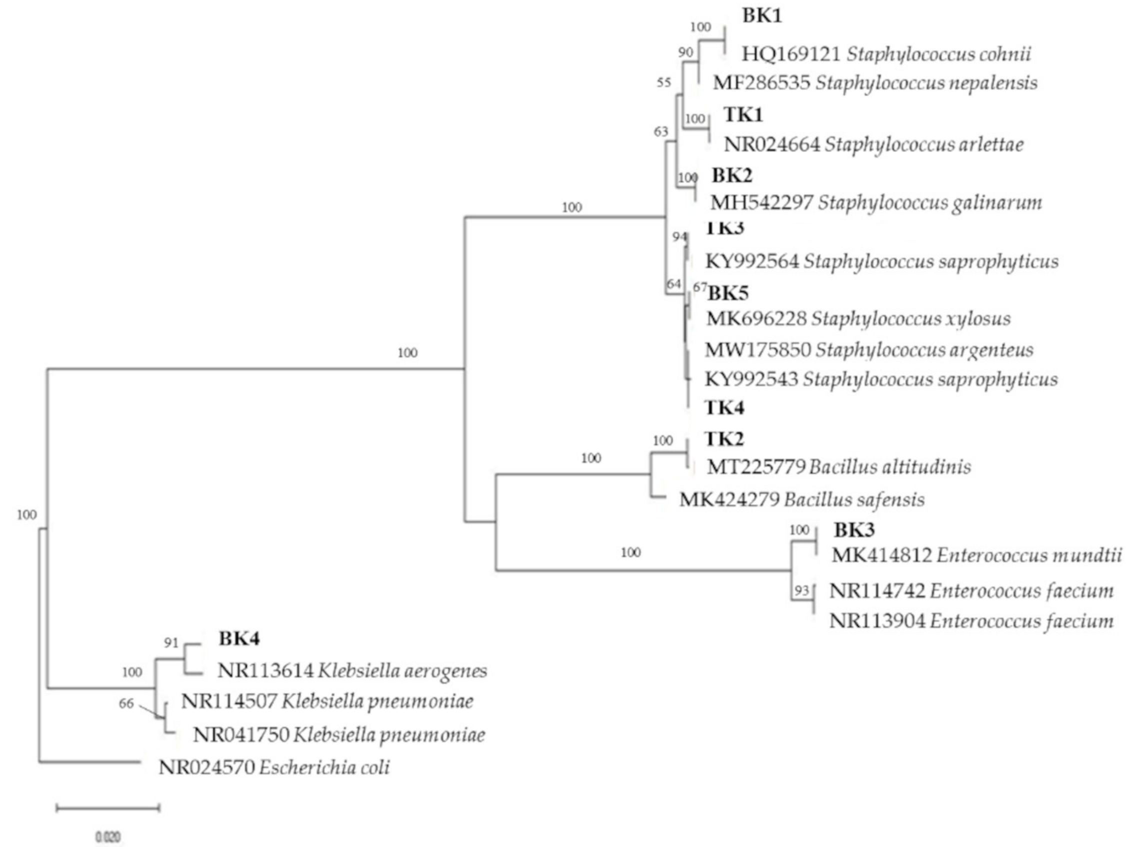

Figure S5. Fungal phylogenetic tree was constructed from sequences from the culturable fungi using MEGA-X with kimura 2 model, the bootstrap values were shown on each branch.

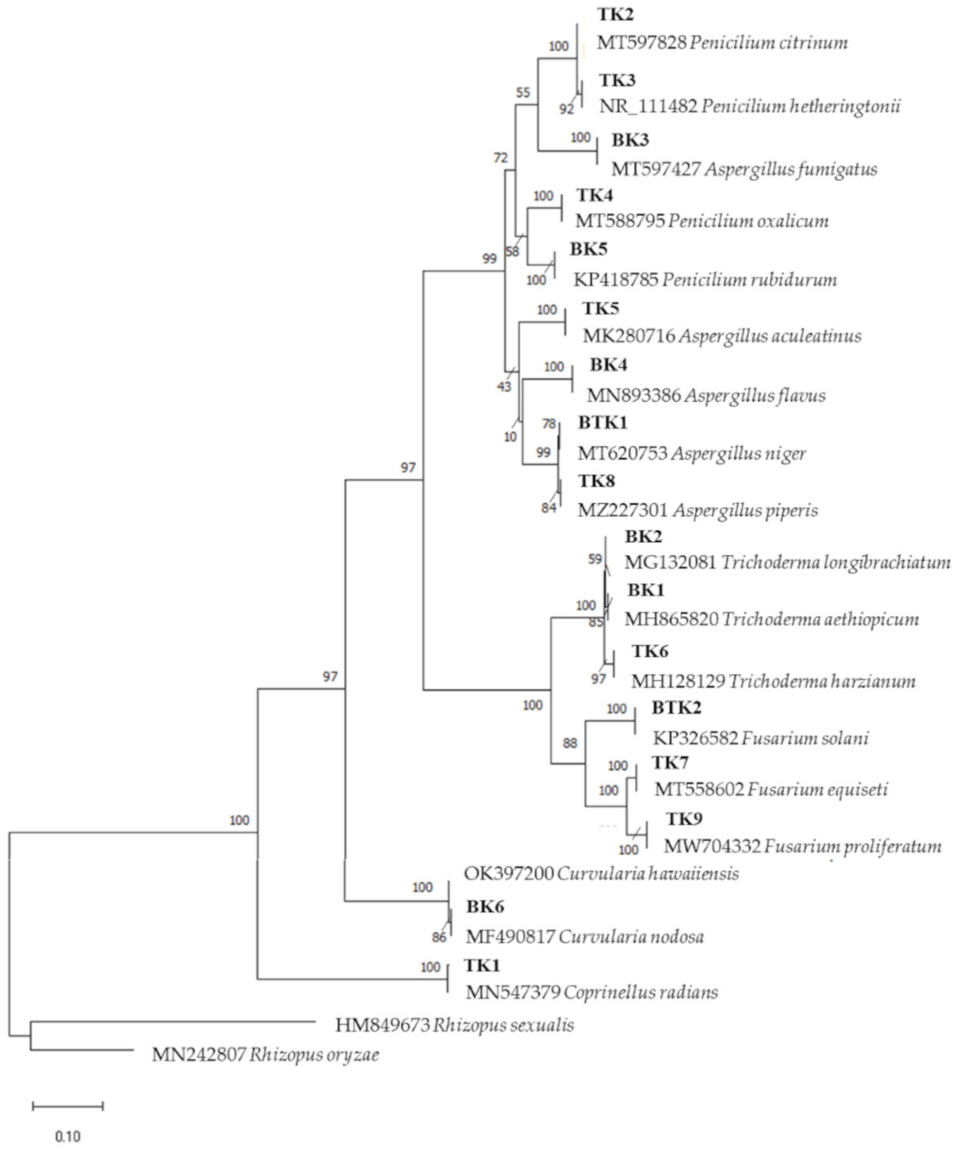

Table S1. Bacterial biodeterioration activity on TSA mixed with crimson red (CR) and malachite green (MG) and fungal biodeterioration activity on PDA mixed with the same color.

Isolate	Code	MG	CR
	Bacteria		
S. cohnii	BK1		
S. galinarum	BK2		
S. xylosus	BK5		
S. arlettae	TK1		
S. argenteus	TK4		
S. saprophyticus	TK3		
K. aerogenes	BK4		
B. altitudinis	TK2		
E. mundtii	BK3		
	Fungi		
T. aethiopicum	BK1		
T. longibrachiatum	BK2		
A.niger	BTK1		
F.solani	BTK2		
A. fumigatus	BK3		
P. citrinum	TK2		
P. hetheringtonii	TK3		
P. oxalicum	TK4		
A..aculeatinus	TK5		
T. harzianum	TK6		
A. piperis	TK8		

Table S2. pH measurement of microorganism isolates, BK; Isolates from Buak Krok Luang temple, TK; Isolates from Tha Kham temple and BTK; Isolates from both temples.

Isolates	Initial pH	Final pH
	Bacteria	
BK3	7.14	7.12 ± 0.04
BK4	7.14	7.15 ± 0.03
TK4	7.14	7.19 ± 0.03
	Fungi	
BK1	5.36	4.41 ± 0.06
BK2	5.36	2.70 ± 0.05
BK3	5.36	3.98 ± 0.06
BTK1	5.36	3.88 ± 0.14
BTK2	5.36	2.81 ± 0.18
TK2	5.36	4.04 ± 0.06
TK3	5.36	4.14 ± 0.07
TK4	5.36	3.33 ± 0.07
TK5	5.36	3.72 ± 0.08
TK6	5.36	4.54 ± 0.11
TK8	5.36	4.16 ± 0.08

Table S3. Comparing between biodeterioration activities from the previously studies and microorganisms from culture-independent method in this study.

Organisms	Biodeterioration process	Researchers
Bacterial		
Cyanobacteria<br>*Synechococcus* sp.,<br>*Chlorogloea* sp.<br>*Chlorella* sp.<br>*Chlorococcum* sp.<br>*Chroococcus* sp.<br>*Gloeocapsa aeruginosa,*<br>*Nostoc muscoru*<br>*Anabaena oscillarioides*<br>*Brasilonema octagenarum*	-Biofilm formation<br>-Creating patinas<br>-Acid production<br>-Rosy discoloration	Hueck, 1965, Sáiz-Jiménez, 1984, Viles, 1987, El-Gorj *et al*., 2019, Imperi *et al*., 2007 and Nugari *et al*., 2009
Arthrobacter parietis<br>*Arthrobacter castelli*<br>*Arthrobacter monumenti*<br>*Arthrobacter pigmenti,*<br>*Arthrobacter tecti*<br>*Arthrobacter tumbae*<br>*Arthrobacter agilis*<br>*Arthrobacter aurescens*<br>*Micrococcus roseuss*	-Rosy discoloration<br>-Biofilm formation	Tescari *et al*., 2018, Heyrman *et al*., 2008 and Altenburgera *et al*., 1996
Fungi		
Cladosporium cladosporioides<br>*Cladosporium oxysporum*<br>*Cladosporium uredinicola*<br>*Cladosporium sphaerospermum*<br>*Cladosporium xylophilum*<br>*Cladosporium macrocarpum*<br>*Cladosporiun herbarum*<br>*Cladosporium tenuisimum*	-Staining<br>-Creating patinas	Sterflinger and Piñar, 2013, Gorbushina *et al*., 2004, Unković *et al*., 2018, Jurado *et al*., 2021, Vukojevi *et al*., 2010

Table S4. Basic data relates to Figure 8. The table represents the microorganisms which are found in each country (dark gray indicates that this study did not focus on the organism).

Researchers	Organisms	
	Bacteria	Fungi
Gorbushina et al., 2004 (Germany)	Gammaproteobacteria<br>Cyanobacteria<br>Firmicutes<br>Actinobacteria	*Aspergillus* sp.<br>*Penicillium* sp.<br>*Cladosporium* sp.<br>*Fusarium* sp.
Tonolo and Giacobini, 1961 (Italy)		*Aspergillus* sp.<br>*Penicillium* sp.<br>*Cladosporium* sp.
Voronina, 1966 (Georgia)		*Aspergillus* sp.<br>*Penicillium* sp.<br>*Cladosporium* sp.<br>*Alternaria* sp.
Kuritzyna, 1968 (Russia)		*Aspergillus* sp.<br>*Penicillium* sp.<br>*Cladosporium* sp.
Unković *et al*., 2016 (Serbia)		*Aspergillus* sp.<br>*Penicillium* sp.<br>*Cladosporium* sp.
Shirakawa *et al*., 2002 (Brazil)		*Aspergillus* sp.<br>*Cladosporium* sp.<br>*Fusarium* sp.<br>*Curvularia* sp.<br>*Alternaria* sp.
López-Miras *et al*., 2013 (Spain)	Gammaproteobacteria<br>Firmicutes<br>Actinobacteria	
Senbua and Wichitwechkam, 2019 (Thailand), Suphaphimol *et al*., 2021 (Thailand)	Gammaproteobacteria<br>Cyanobacteria<br>Firmicutes<br>Actinobacteria	*Aspergillus* sp.<br>*Penicillium* sp.<br>*Fusarium* sp.<br>*Curvularia* sp.<br>*Neodevriesia* sp.
Duan *et al*., 2018 (China), Duan *et al*., 2019 (China), Ma *et al*., 2020 (China)	Gammaproteobacteria<br>Cyanobacteria<br>Firmicutes<br>Actinobacteria	*Aspergillus* sp.<br>*Penicillium* sp.<br>*Cladosporium* sp.<br>*Fusarium* sp.<br>*Alternaria* sp.<br>*Mucor* sp.<br>*Malassezia* sp.<br>*Curvularia* sp.

Nikola *et al.*, 2018 (Serbia)		*Aspergillus* sp.<br>*Penicillium* sp.<br>*Cladosporium* sp.
